# Pan-cancer analysis of non-coding recurrent mutations and their possible involvement in cancer pathogenesis

**DOI:** 10.1093/narcan/zcab008

**Published:** 2021-03-22

**Authors:** Chie Kikutake, Minako Yoshihara, Mikita Suyama

**Affiliations:** Medical Institute of Bioregulation, Kyushu University, Fukuoka 812-8582, Japan; Medical Institute of Bioregulation, Kyushu University, Fukuoka 812-8582, Japan; Medical Institute of Bioregulation, Kyushu University, Fukuoka 812-8582, Japan

## Abstract

Cancer-related mutations have been mainly identified in protein-coding regions. Recent studies have demonstrated that mutations in non-coding regions of the genome could also be a risk factor for cancer. However, the non-coding regions comprise 98% of the total length of the human genome and contain a huge number of mutations, making it difficult to interpret their impacts on pathogenesis of cancer. To comprehensively identify cancer-related non-coding mutations, we focused on recurrent mutations in non-coding regions using somatic mutation data from COSMIC and whole-genome sequencing data from The Cancer Genome Atlas (TCGA). We identified 21 574 recurrent mutations in non-coding regions that were shared by at least two different samples from both COSMIC and TCGA databases. Among them, 580 candidate cancer-related non-coding recurrent mutations were identified based on epigenomic and chromatin structure datasets. One of such mutation was located in RREB1 binding site that is thought to interact with *TEAD1* promoter. Our results suggest that mutations may disrupt the binding of RREB1 to the candidate enhancer region and increase *TEAD1* expression levels. Our findings demonstrate that non-coding recurrent mutations and coding mutations may contribute to the pathogenesis of cancer.

## INTRODUCTION

Cancer is a disease that involves dysregulation of cell cycle progression and abnormal cell growth. Cells accumulate multiple somatic mutations during their lifetime due to various factors, such as smoking, drinking and exposure to UV radiation, which contribute to the development of cancer. Among the different types of genetic mutations, those directly involved in cancer development and progression are classified as driver mutations and, as such, many studies have examined their functions in cancer cells (1). Elucidation of the role of driver mutations is important for their effective use as not only diagnostic and prognostic markers, but also pharmacologic targets. Based on accumulated knowledge on driver mutations, clinical sequencing has become widespread in the medical field, providing optimal treatments for mutation patterns (2).

However, only about 20% of cancer patients receive FDA-approved drugs that are more effective than the standard treatment (3). One reason for this limitation is that there are cancer-related mutations that have not yet been identified and that corresponding treatments have not been well developed. The primary targets of cancer research have been mutations in the coding regions of genes, which comprise about 2% of the whole genome. Studies have identified ∼500 cancer-related genes (4,5). However, little is known about cancer-related mutations in non-coding regions of the genome (6–8). The most well-known non-coding mutations are those in *TERT* gene promoter (9). These mutations induce telomerase re-expression, which implies that cells that were originally destined to die via apoptosis can continue to undergo cell division. Therefore, mutations in non-coding regions can also be potential cancer drivers.

In the past decade, more cancer genome data have been deposited in public databases as a result of the development of next-generation sequencing technologies. Until recently, most of these cancer genome data were derived from exome sequencing; however, in the past few years, the amount of whole-genome sequencing (WGS) data has been increasing (10). These new data have made it possible to analyze mutations in non-coding regions of the genome.

Interpretation of mutations in non-coding regions has been challenging. While mutations in coding regions can easily be annotated for their cellular impacts based on the associated changes in the amino acid sequence, annotating non-coding mutations is less straightforward due to the difficulty in elucidating their associated functions. In addition, there is a huge number of mutations in non-coding regions of the genome. Recently, the increasing amount of epigenomic data, such as those from IHEC database (11), ENCODE database (12) and ChIP-Atlas (13), has enabled the interpretation of whole-genome sequences, including those of non-coding regions.

In this study, to comprehensively identify cancer-related non-coding mutations, we focused on recurrent mutations in non-coding regions using WGS data on different cancer samples. Regions with such recurrent mutations are thought to have some influence on cancer development, since the likelihood of mutations occurring by chance at the same position in the genome is statistically low. We used non-coding variant data from COSMIC (14) and WGS data from TCGA (15) to extract 21 574 non-coding recurrent mutations. Among these, we identified 580 candidate cancer-related non-coding recurrent mutations (CNRMs) based on characteristics that are suggestive of enhancer mutations. Of the identified mutations, we annotated three possible enhancer mutations using various epigenomic data.

## MATERIALS AND METHODS

### Datasets

Non-coding variants were downloaded from the COSMIC data repository (https://cancer.sanger.ac.uk/cosmic/CosmicNCV.tsv.gz) (GRCh38, release v89; 15 May 2019) (14). The ‘non-coding variants’ is one of the somatic mutation datasets in COSMIC data repository. These data are manually curated non-coding somatic mutation information that relates to human cancers. Exonic regions were defined by Homo_sapiens.GRCh38.96.gtf from Ensembl (https://asia.ensembl.org/Homo_sapiens/Info/Index) (16). Then, coordinates of the mutations extracted from the COSMIC data were converted from human genome assembly GRCh38 to GRCh37 using the liftOver program (17). WGS data of paired normal and tumor tissues (*n* = 930) of 23 cancer types (Table 1) in TCGA (18) were downloaded from http://ideker.ucsd.edu/papers/wzhang2017/ (15). Only data from solid tumors were used (*n* = 891). We also downloaded clinical data, RNA-seq data and ATAC-seq data (https://gdc.cancer.gov/about-data/publications/ATACseq-AWG) (19) from TCGA. The ATAC-seq data were derived from 404 samples in 23 cancer types (Table 1). Chromatin interaction data including 3C, 4C, 5C, ChIA-PET, Hi-C and IM-PET were downloaded from 4DGenome (https://4dgenome.research.chop.edu/) (20). Transcription factor binding sites (TFBSs) were adopted if the associated score in the JASPAR database was ≥400 (http://jaspar.genereg.net/) (21). The human reference genome GRCh37 was used in this study.

**Table 1.** The sample sizes of WGS, ATAC-seq and RNA-seq from TCGA

Cancer types	Abbreviation	WGS	ATAC-seq	RNA-seq
Adrenal gland	ACC		9	
Bladder urothelial carcinoma	BLCA	23	10	23
Breast adenocarcinoma	BRCA	99	74	98
Cervical squamous cell carcinoma and endocervical adenocarcinoma	CESC	20	2	20
Cholangiocarcinoma	CHOL		5	
Colon and rectal adenocarcinoma	COAD, READ	65	38	61
Esophageal carcinoma	ESCA		18	
Glioblastoma multiforme	GBM	52	9	34
Head and neck squamous cell carcinoma	HNSC	50	9	49
Kidney chromophobe	KICH	49		49
Kidney renal clear cell carcinoma	KIRC	41	16	41
Kidney renal papillary cell carcinoma	KIRP	36	34	33
Lower grade glioma	LGG	19	13	19
Liver hepatocellular carcinoma	LIHC	54	17	52
Lung adenocarcinoma	LUAD	50	22	49
Lung squamous cell carcinoma	LUSC	50	16	48
Mesothelioma	MESO		7	0
Ovarian serous cystadenocarcinoma	OV	50		24
Pheochromocytoma and paraganglioma	PCPG		9	
Prostate adenocarcinoma	PRAD	20	26	20
Sarcoma	SARC	34		34
Skin cutaneous melanoma	SKCM	38	13	38
Stomach adenocarcinoma	STAD	40	21	35
Testicular germ cell tumor	TGCT		9	
Thyroid carcinoma	THCA	50	14	48
Uterine corpus endometrial carcinoma	UCEC	51	13	50

### Screening of candidate CNRMs

**Figure 1.**
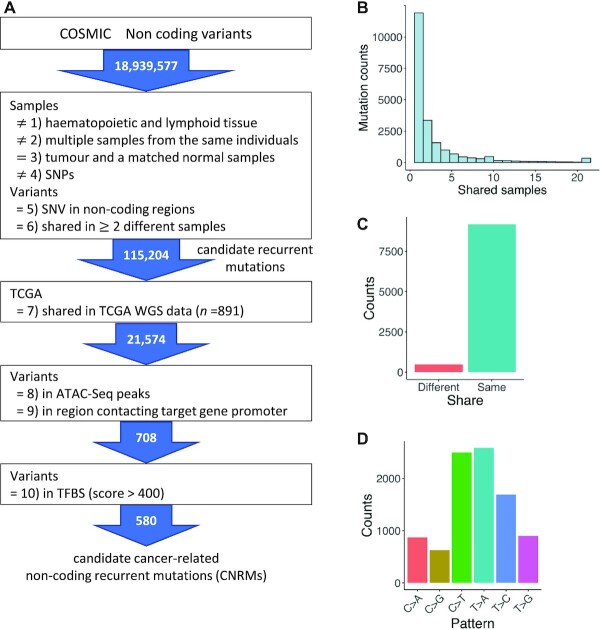
Overview of identification of recurrent mutations in non-coding regions and characteristics of the extracted mutations. (**A**) Workflow used to identify candidate CNRMs in this study. Using the filtering criteria (shown in the boxes), recurrent mutations in non-coding regions were extracted from a non-coding variant dataset in COSMIC. The numbers in arrows represent the number of extracted mutations. A number followed by a closing parenthesis corresponds to that of the filtering criterion in the main text. (**B**) Distribution of 21 574 mutation sites in TCGA samples. The horizontal axis shows the number of recurrences and the vertical axis represents the number of mutation positions. For example, height of the bar at the value 2 in the horizontal axis indicates the number of mutations shared by two samples. (**C**) Bar plot showing the distribution of mutational patterns of 9657 recurrent mutations shared by ≥2 samples. ‘Different’ indicates the recurrent mutations in which the mutational patterns were not identical among the samples, while ‘same’ indicates those that were identical. (**D**) The frequency of the six mutational patterns for mutations classified as ‘same’.

To explore recurrent mutations in the non-coding regions, we applied several filtering criteria to the COSMIC data on the non-coding variants. The filtering criteria for mutations (Figure 1A) were as follows: (1) mutations obtained from hematopoietic and lymphoid tissue samples were removed; (2) mutations obtained from multiple samples from the same individuals were removed; (3) somatic mutations obtained by sequencing both tumor and matched normal samples from the same patient were extracted; (4) mutations flagged as single nucleotide polymorphisms (SNPs) in the COSMIC database were filtered out; (5) mutations located in exonic regions were removed; and (6) mutations shared among at least two different samples were extracted as recurrent mutations. Among them, we focused only on mutations shared in at least two cancer types. This criterion was applied to remove sequencing and mapping artifacts (22). According to previous studies, some mutations are likely to occur in specific sequences depending on specific cancer types, such as mutation due to damage from ultraviolet light or impaired nucleotide excision repair at sites occupied by transcription factors (TFs) in melanoma. In the case of mutations that occur in same position in the same cancer type, it is possible that the mutations are associated with the localized mutational process.

To extract recurrent mutations which is potentially involved in cancer, we also analyzed the corresponding mutations in WGS data from TCGA, which has various associated data, such as clinical data and RNA-seq data. We applied the following filtering criteria: (7) extracting mutations in WGS data from TCGA, which are located in the same position as those extracted from COSMIC data in no. (6) described above; (8) extracting mutations located in open chromatin regions using ATAC-seq data; (9) extracting mutations located in chromatin-interacting regions using 4DGenome data; and (10) extracting mutations located in TFBSs using JASPAR data.

### Evaluation of the potential impact of mutations on TFBS sequences

To assess the effect of a mutation on TFBS sequence, we calculated the difference in Shannon entropy *C* between reference and alternate nucleotides (23,24). The Shannon entropy ${C_i}$ of the *i*th site in the sequence was calculated using the following formula:

$$\begin{equation*}{\rm{\;}}{C_i} = \;2 + \mathop \sum \limits_{b \in \left\{ {{\rm{A}},{\rm{C}},{\rm{G}},{\rm{T}}} \right\}} {p_{b,i}}{\rm{lo}}{{\rm{g}}_2}{p_{b,i}}\end{equation*}$$

where ${p_{b,i}}$ is the frequency of nucleotide *b* at the *i*-th site. When ${p_{b,i}}\;$is equal to 0, ${\rm{lo}}{{\rm{g}}_2}{p_{b,i}}$ was also regarded as 0. For TFBS with ≥ 2 types of mutations, the Shannon entropy (bit) was calculated for all mutations found in TFBS and the average value of the difference in Shannon entropy was calculated as ‘average Δbit’.

To calculate the expected average Δbit, mutations found in TFBSs were extracted from the ‘non-coding variants’ in COSMIC. Among them, 100 mutations were randomly extracted and the average Δbit in Shannon entropy to TFBSs was calculated. After repeating this calculation 1000 times, the expected average Δbit in Shannon entropy for randomly selected mutations was calculated as −0.25.

### Analysis of gene expression associated with non-coding mutations

To examine the association between recurrent mutations and target gene expression levels, differential expression analysis was performed using 825 RNA-seq data from TCGA. Expression datasets were downloaded from cBioPortal (https://www.cbioportal.org/) (25). We compared the expression levels between the samples with and without mutations in TFBSs that harbor recurrent mutations. More precisely, differential expression analysis was performed on all samples with mutations in each of the TFBSs regardless of whether they are recurrent mutations or not because any mutation in the regulatory elements can affect the expression levels of the target genes. Due to the small sample size, expression levels from different cancer types were integrated using *z*-scores (8) and Wilcoxon rank-sum test was used to evaluate the difference in expression levels.

### Statistical analysis

Statistical analysis was performed using R software version 3.6.1 (R Project for Statistical Computing, Vienna, Austria). For comparisons of two groups, Wilcoxon rank-sum test was adopted. Benjamini–Hochberg (BH) procedure was used to adjust for multiple testing (26). *P*-value < 0.1 and adjusted *P*-values (false discovery rate, FDR) < 0.25 were considered statistically significant in accordance with a previous study (8).

## RESULTS

### Recurrent mutation screening in non-coding elements

To screen candidate CNRMs, we used 18 939 577 non-coding mutation positions registered in COSMIC (14) (Figure 1A). In this study, we focused only on solid tumors because the characteristics of mutations differ between solid and blood tumors (27). We extracted somatic mutations by sequencing of both tumor and matched normal samples from the same patient. To further exclude possible germline SNPs, we filtered out mutations that were flagged as SNPs in COSMIC. This excluded 22.5% of the somatic mutations as SNPs. Then, we excluded mutations located in exonic regions using human gene annotation from Ensembl (release 96). Of these extracted mutations, those found in the same positions in at least two different samples were defined as candidate recurrent mutations (115 204 mutations) and were used for further study (Figure 1A). We extracted the corresponding mutations in WGS data from TCGA, in which there are various associated data for each sample as well as mutation data. Of the 115 204 candidate recurrent mutation positions found in COSMIC, 21 574 mutation positions existed in WGS in TCGA (Figure 1A and B; Supplementary Table S1).

To confirm whether the recurrent mutations are enriched in non-coding regions, we analyzed the difference of recurrent mutation distribution between the coding region and the non-coding region using the WGS data in TCGA. Among them, 4.1% were found in the coding region. Comparing the value to the total length of the coding region (2.8%), mutations in the WGS data were significantly enriched in the coding region (*P* < 0.001).

Among the 21 574 mutation positions from TCGA data, the highest recurrence was exhibited by the mutation at chr3:75 844 229, which was shared by 71 independent samples and located in about 10 kb upstream of *ZNF717*, although it is not recognized as a cancer-related gene. Other highly recurrent sites were observed in chr5:1 295 228 and chr5:1 295 250, both of which are located in *TERT* promoter. These mutations were shared by 69 and 29 independent samples, respectively. Changes in cellular function by mutations in *TERT* promoter region have been previously reported (9). Since these mutations (in the *TERT* promoter) were extracted using the non-coding mutation filtering criteria in this study, the validity of this procedure was reinforced.

We next examined each recurrent mutation for consistency in the mutation pattern. Among the 21 574 positions, 9657 were shared in ≥2 samples. Of the 9657 positions, 9176 (95.0%) showed identical substitution patterns for each recurrent mutation (Figure 1C). In these 9176 positions of recurrent mutations, we analyzed the mutational pattern (Figure 1D). The most common patterns of alterations were T > A transversion and C > T transition, which was observed in 2584 (28.2%) and 2499 (27.2%) recurrent mutations, respectively. For comparison of the mutation pattern in non-coding recurrent mutations with all the mutation in coding and non-coding regions, we counted the mutations of each mutation pattern (Supplementary Figure S1A and B). Among the coding and non-coding mutations from WGS data in TCGA, the most common pattern of alteration was also C > T transition. Generally, C > T transition dominates the mutation pattern in many cancer types (28) and are generated for various reasons, such as UV exposure, failure of DNA mismatch repair and spontaneous deamination of CpG dinucleotides (14).

To extract functionally relevant mutations from the identified recurrent mutations, we ascertained whether these mutations were located within *cis*-regulatory elements by comparing them with open chromatin regions using a genome-wide ATAC-seq dataset from 23 cancer types in TCGA (19). ATAC-seq can reveal open chromatin regions, especially those predicted to have TFBSs (29). Among the 21 574 mutations observed in WGS data in TCGA, we identified 1722 mutations located in the open chromatin regions defined by ATAC-seq data.

To assess whether these mutations are enriched in open chromatin regions, we randomly extracted 10 000 mutations from the non-coding variant data in TCGA. After repeating the calculation 10 000 times, the expected proportion of the non-coding variants found in open chromatin regions was calculated as 8.04%. On the other hand, in the case of the 21 574 mutation positions in TCGA, it was calculated as 7.98%. The result indicates that recurrent mutations in the non-coding regions were not significantly enriched in the open chromatin regions (*P* = 0.762). On average, 0.012 mutations per sample were found per ATAC-seq peak, with at least one recurrent mutation. In contrast, on average, 0.0073 mutations per sample were found per ATAC-seq peak, without recurrent mutation. The number of mutations in open chromatin (with at least one recurrent mutation) was significantly higher than those without recurrent mutations (*P* = 0.0040).

Furthermore, to extract mutations in spatially interacting chromatin regions, we used 4DGenome data (20). This public database comprises information from both experimental studies (3C, 4C, 5C, Hi-C, ChIA-PET and Capture-C) and computational predictions (IM-PET). Among the 1722 mutations, we identified 708 mutations located in spatially interacting chromatin regions defined by 4DGenome data. When 4Dgenome data are applied to the 21 574 mutations, 3986 mutations were located in the spatially interacting chromatin regions. The 708 mutations correspond to 41.1% (708/1722) of mutations identified with the ATAC-seq data and 17.8% (708/3986) of mutations identified with the 4DGenome data.

Finally, to further narrow down the 708 mutations to which TFs are likely to bind, we also selected only those mutations that were located in TFBSs predicted by JASPAR motif data (2018 release, score ≥ 400) (21). These filtrations resulted in 580 candidate CNRMs (Figure 1A).

### Recurrent mutations in enhancer regions

**Figure 2.**
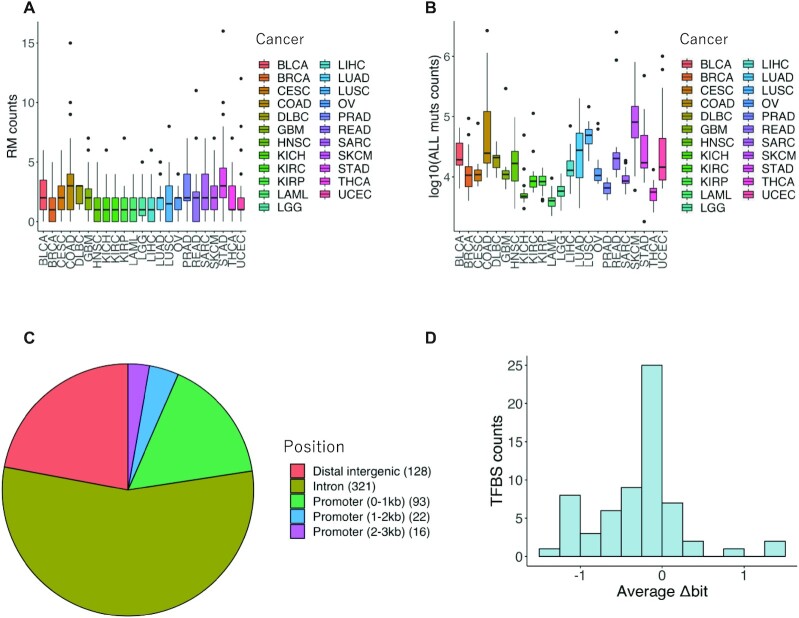
Characteristics of candidate CNRMs. (**A**) Box plot showing the number of CNRMs for each cancer type. (**B**) Box plot showing the number of all mutations for each cancer type. Note that the vertical axis is in logarithmic scale. (**C**) Distribution of distance for 580 candidate CNRMs from the TSS of the nearest gene. (**D**) Distribution of average Δbit for 64 TFBSs.

For these 580 candidate CNRMs, we counted the number of CNRMs per sample for each of the 23 cancer types (Figure 2A). We also counted the total number of mutations per sample for each cancer type (Figure 2B). The total number of mutations for some cancer types was highly variable, such that higher numbers of mutations were observed in melanoma and colorectal cancer (Figure 2B), while the number of CNRMs was rather uniform among cancer types (Figure 2A). This indicates that passenger mutations could have been largely excluded from CNRMs. However, this does not necessarily mean that all identified CNRMs are driver mutations. We further examined the position of the extracted 580 mutations in terms of gene structure. The largest number of CNRMs was observed in introns (55.3%) (Figure 2C), although the combined length of all intronic regions account for approximately 25% of the entire genome (30). The proportion of CNRMs in introns (55.3%) was supposed to be a reasonable value because the proportion of the total length of the open chromatin region existing in the intronic region was 63.8%, which was calculated from the ATAC-seq data obtained from TCGA (19). Furthermore, to examine whether the 580 CNRMs identified in this study are located in miRNA target sites, we investigated overlaps between miRNA target sites registered in ORegAnno database (31) and CNRMs. We found that no CNRM was located in these miRNA target sites.

To examine whether the 580 CNRMs could have impacts on TFBSs for their enhancer activity, we extracted 3731 TFBSs with CNRMs using JASPAR data. The increase in the number of TFBSs from the 580 CNRMs is as a result of the existence of overlapping TFBSs for a single CNRM. We compared the expression levels of their 1104 target genes, which were predicted to spatially contact with TFBSs in 4DGenome data, between samples with, and without mutations. Here, we focused on 727 TFBSs that harbored ≥5 mutations in distinct samples to retain statistical power. Among these 727 TFBSs, 64 showed significant differences in expression levels of the target genes (FDR < 0.25) (Supplementary Table S2), indicating that those mutations could affect their enhancer activity. To confirm the validity of FDR < 0.25 for gene expression analysis, we performed a simulation analysis. First, we randomly extracted 1000 genes included in 4DGenome data. Then, of the 825 samples with associated RNA-seq data in TCGA, we randomly selected 38 samples as those assumed to have mutations and the remaining 787 samples as those assumed to be wild-type (WT). In this simulation, we used 38 samples as those assumed to have mutations because the maximum number of samples with mutations in Supplementary Table S2 is 38. Then, we compared the gene expression levels between samples assumed to have mutations and those assumed to be WT. After repeating the above procedure 100 times, we detected on average 0.39 genes with FDR < 0.25 among the randomly selected 1000 genes. Therefore, the possibility that a gene accidentally selected as a difference in expression level is extremely low, with FDR < 0.25.

The cancer-related genes *BCL6, FANCC, PICALM* and *SGK1* were included among the target genes of the 64 TFBSs (14). We also evaluated the impact of the mutations on the 64 TFBS motifs by calculating the difference in Shannon entropy (Δbit) (Figure 2D and Supplementary Table S2). From the distribution of the difference in Shannon entropy, we found that the number of mutations that had negative values, especially those that created weaker TFBS motifs, were higher than those that created stronger motifs.

To examine whether the 1104 target genes are included in the 151 target genes for the non-coding driver elements identified by Rheinbay *et al.* (22), we compared these gene lists. The results showed that only eight genes (*WDR74, INTS4, PRDX2, SLC12A5, EN1, MLXIPL, RNF121* and *HOXB5*) were shared in both gene lists. Furthermore, in the case of the target genes for 64 TFBSs, which showed significant differences in expression levels, only two genes (*EN1* and *SLC12A5*) were shared in both gene lists. We found only a slight overlap between the results of Rheinbay *et al.* and our results probably because of the differences in definition of the regulatory elements. Rheinbay *et al.* (22) analyzed the functional elements defined by GENCODE and other annotations such as miRNA and lncRNA. Most of these elements were located close to gene bodies such as promoters and UTRs. In contrast, in this study, we defined functional elements mainly by chromatin structures and TFBSs.

### Functional interpretation of the candidate CNRMs within TFBSs

In the following sections, we selected three TFBSs with candidate CNRMs and interpreted them with epigenomic data and chromatin structural data. The association between CNRMs and target gene expression levels were also evaluated. In addition, for each TFBS, we examined the publicly available ChIP-seq data for histone modifications and TF bindings.

### Recurrent mutations identified in *TEAD1* enhancer

**Figure 3.**
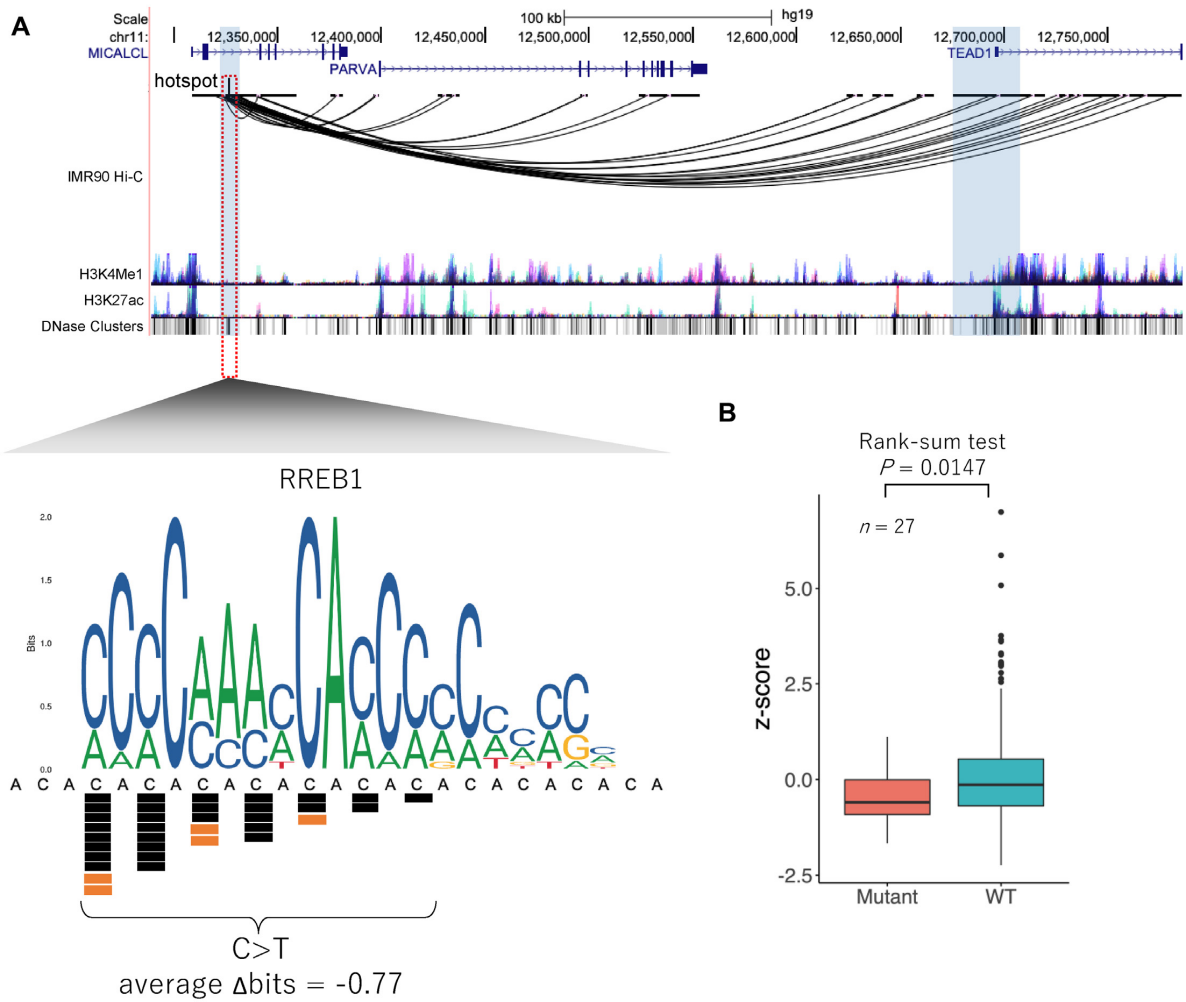
Recurrent mutations identified in *TEAD1* enhancer. (**A**) The region including the recurrent mutations is in an intron of *MICALCL* gene. Hi-C chromatin structure in IMR90 cells, two kinds of histone marks, and DNase I hypersensitive sites are shown. The lower panel shows a close-up view of the genomic region surrounding the recurrent mutations. Mutations from TCGA data (black squares), COSMIC data (orange squares) and sequence motif of RREB1 binding are shown. (**B**) *TEAD1* expression levels of samples with and without mutations in the RREB1 binding site. A Wilcoxon rank-sum test was used to compare the expression levels between the samples with and without mutations (red and green, respectively).

Within the RREB1 binding site located at chr11:12 326 201–12 326 221, we found a total of 29 mutations at seven unique positions in TCGA samples and five mutations at three unique positions in COSMIC samples. Analysis of chromatin interaction by Hi-C data from IMR90 cells and human mammary epithelial cells (HMECs) showed that this region of chr11:12 326 201–12 326 221 interacts with *TEAD1* (Transcriptional enhancer activator domain 1) promoter region (Figure 3A and Supplementary Figure S2). These mutations were located at relatively conserved cytosine nucleotides in the RREB1 binding site. Although the binding of RREB1 to this region could not be confirmed due to lack of the ChIP-seq data, the region was located at a DNase I hypersensitive site (Figure 3A). TF RREB1 has been previously reported to function as both an activator and a repressor of cell growth, cell differentiation, transcriptional regulation and DNA damage repair (32). RREB1 also plays an important role in Ras signaling, leading to the development of several cancers (33).

To examine the influence of the mutations in this TFBS motif in the candidate enhancer region on the expression of *TEAD1*, we used 27 RNA-seq datasets containing at least one mutation in RREB1 binding site from TCGA. These mutations were observed in several cancer types (BRCA; *n* = 5, COAD; *n* = 3, GBM, *n* = 2; KICH, *n* = 1; KIRC, *n* = 1; KIRP, *n* = 1; LGG, *n* = 2; LIHC, *n* = 1; LUAD, *n* = 2; LUSC, *n* = 2; STAD, *n* = 5; THCA, *n* = 2). In addition, we compared the expression levels of *TEAD1* in these samples to those without mutations. To compare the expression levels among different cancer types, expression levels were normalized to *z*-scores. We found that the expression levels of *TEAD1* were significantly decreased in samples with these mutations (*P*-value = 0.0147) (Figure 3B). In addition, the same analysis was also applied to four other neighboring genes of *TEAD1* locus to confirm that the effect of the mutations was specific for *TEAD1*. The results showed no significant differences in the expression levels of these four genes, indicating that the target gene of the RREB1 motif is likely to be *TEAD1* (Supplementary Figure S3).

### Recurrent mutations identified in *CX3CR1* enhancer

**Figure 4.**
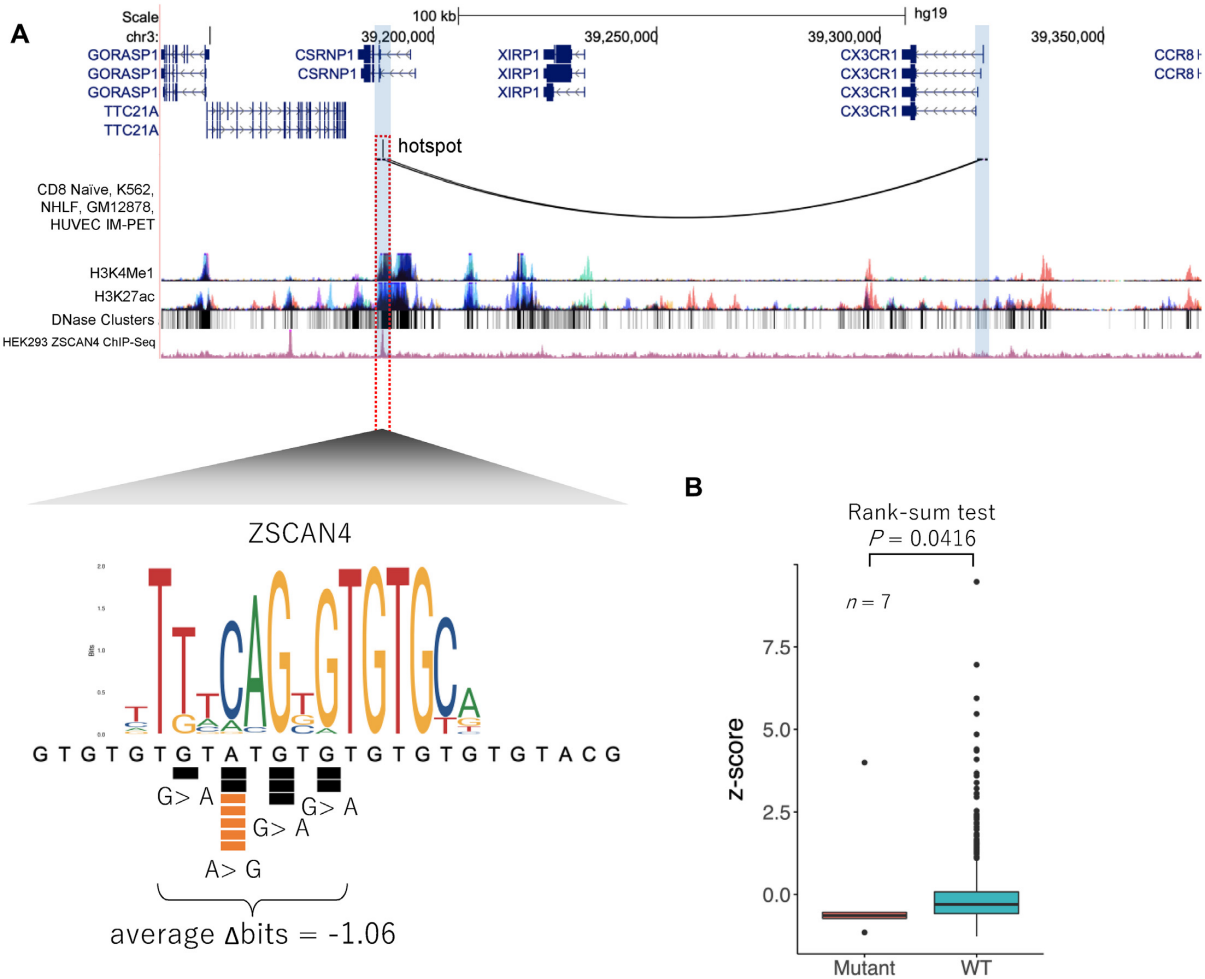
Recurrent mutations identified in *CX3CR1* enhancer. (**A**) The region including the recurrent mutations is located in an intron of *CSRNP1* gene. IM-PET data, two kinds of histone marks and DNase I hypersensitive sites are shown. Below these tracks, ChIP-seq peaks of ZSCAN4 from HEK293 are shown. The lower panel shows a close-up view of the genomic region surrounding the recurrent mutations. Mutations from TCGA data (black squares), COSMIC data (orange squares) and sequence motif of ZSCAN4 binding are shown. (**B**) *CX3CR1* expression levels of samples with and without the mutation in the ZSCAN4 binding site. A Wilcoxon rank-sum test was used to compare the expression levels between the samples with and without mutations (red and green, respectively).

Eight independent samples from TCGA data and five independent samples from COSMIC data had mutations within chr3:39 188 723–39 188 738, which is located at relatively highly conserved nucleotides in ZSCAN4 binding site in an intron of *CSRNP1* gene. IM-PET data from 4DGenome from CD8 Naïve, K562, NHLF, GM12878 and HUVEC cells and Hi-C data from HMECs showed that this region interacts with *CX3CR1* (C-X3-C Motif Chemokine Receptor 1) promoter (Figure 4A and Supplementary Figure S4). ChIP-seq data from HEK293 cells demonstrate that ZSCAN4 actually binds to this region (Figure 4A).

To investigate the relationship between mutations in the candidate enhancer region and expression levels of *CX3CR1*, we compared the expression levels of *CX3CR1* in samples with the mutations to those without the mutations. These mutations were observed in seven samples (BRCA, *n* = 3; HNSC, *n* = 1; LUAD, *n* = 1; THCA, *n* = 1; UCEC, *n* = 1) in TCGA with RNA-seq data. We found that the expression levels of *CX3CR1* were significantly reduced in samples with the mutations (*P*-value = 0.0416) (Figure 4B). The same analysis was also applied to four other neighboring genes of the *CX3CR1* locus to confirm that the effect of the mutations in the ZSCAN4 motif was specific for *CX3CR1*. The results indicated no significant differences in the expression levels of these genes (Supplementary Figure S5). Therefore, the mutations in the ZSCAN4 binding site would specifically affects the expression level of *CX3CR1*.

### Recurrent mutations identified in *NFYB* enhancer

**Figure 5.**
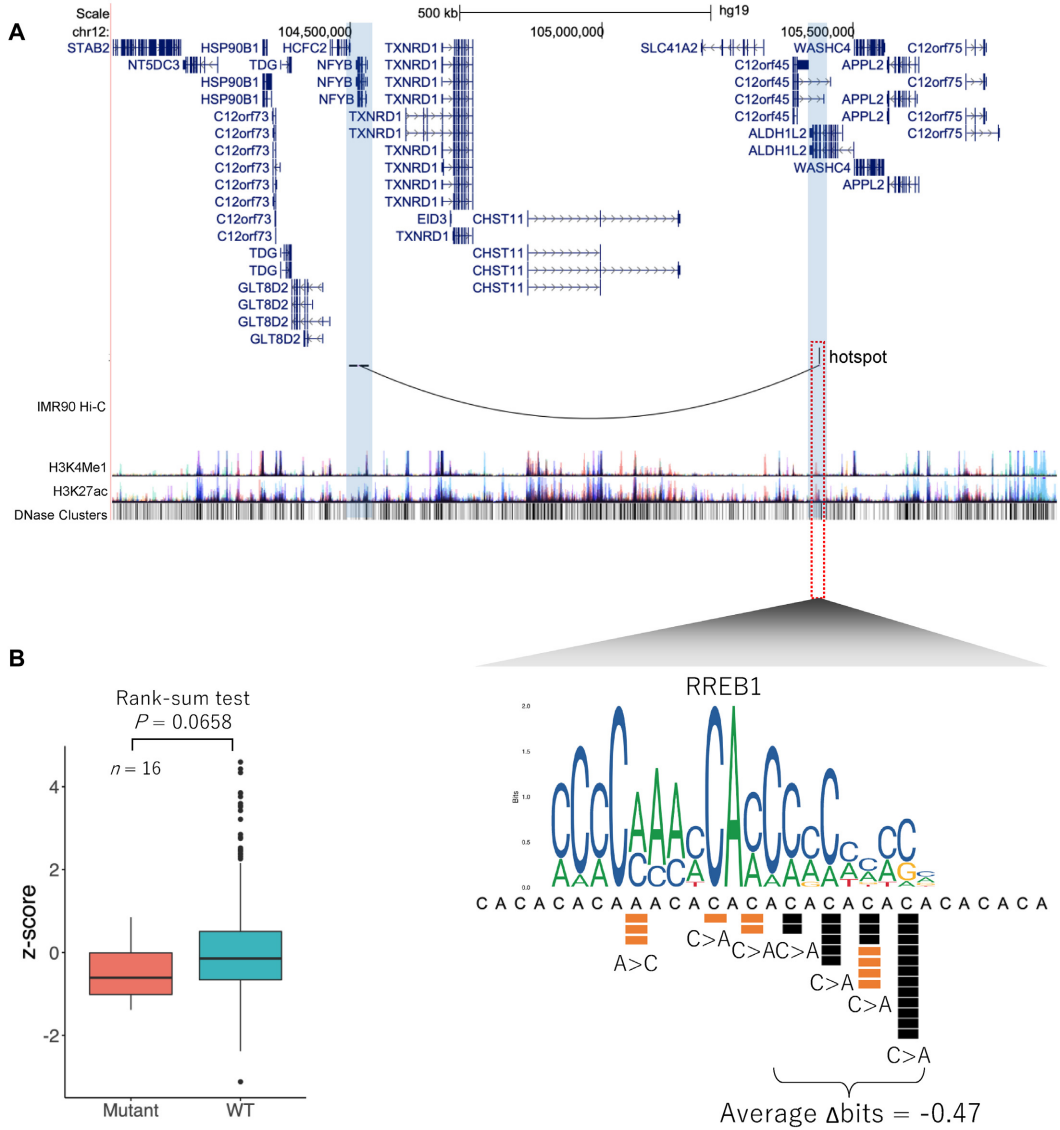
Recurrent mutations identified in *NFYB* enhancer. (**A**) Recurrent mutations found in an intron of *C12orf45* and *ALDH1L2* genes. The gene tracks are followed by Hi-C interactions in IMR90 cells, histone modifications and DNase I hypersensitive sites. The lower panel shows a close-up view of the genomic region surrounding the recurrent mutations. Mutations from TCGA data (black squares), COSMIC data (orange squares), and sequence motif of the RREB1 binding site are shown. (**B**) *NFYB* expression levels of samples with and without the mutation in the RREB1 binding site. A Wilcoxon rank-sum test was used to compare the expression levels between the samples with and without mutations (red and green, respectively).

One of the CNRMs was observed within chr12:105 432 052–105 432 072. In the region, 22 independent samples from TCGA data and 10 independent samples from COSMIC data had mutations. These recurrent mutations were located in an intron of *C12orf45* and *ALDH1L2* genes (Figure 5A) and at relatively conserved cytosine nucleotides in RREB1 binding site, which was found in a DNase I hypersensitive site (Figure 5A). It is suggested to interact with *NFYB* (Nuclear Transcription Factor Y Subunit Beta) promoter according to Hi-C data from IMR90 cells and HMECs (Figure 5A and Supplementary Figure S6).

To investigate the relationship between mutations in the candidate enhancer region and expression levels of *NFYB*, we used 16 RNA-seq data registered in TCGA (GBM, *n* = 1; HNSC, *n* = 1; KICH, *n* = 2; KIRC, *n* = 1; LIHC, *n* = 1; LUAD, *n* = 1; LUSC, *n* = 1; SARC, *n* = 3; SKCM, *n* = 1; STAD, *n* = 1; THCA, *n* = 2; UCEC, *n* = 1), which have the mutations in TFBS. The expression levels of *NFYB* in these 16 samples having the mutations were compared to those without the mutations. The results showed that the expression levels of *NFYB* in the samples with the mutations were significantly lower than those without the mutations (*P*-value = 0.0658) (Figure 5B). In addition, the same analysis was also applied to 10 other neighboring genes of *NFYB* to confirm that the effect of the mutations was specific for *NFYB*. No significant differences were observed in the expression levels of those genes, indicating that the target gene of the motif is likely *NFYB* (Supplementary Figure S7).

## DISCUSSION

In this study, we focused on non-coding recurrent mutations observed at identical positions in the genome of different cancer samples in order to identify novel cancer-related mutations. We analyzed recurrent mutations because the probability that a mutation occurs by chance at the same position in different samples is extremely low, considering the length of the whole genome. Therefore, we hypothesized that these non-coding recurrent mutations may have some undefined influence on cancer, such as alteration of gene expression. We searched for recurrent mutations using public dataset of ‘non-coding variants’ in COSMIC and applied several filters to identify candidate CNRMs in enhancer regions that may have an important role in the pathogenesis of cancer.

After screening for non-coding variants, we identified 21 574 mutations in WGS data from TCGA that were shared among at least two different samples in COSMIC data. Among them, we successfully identified 580 candidate CNRMs. We then focused on three TFBSs with the CNRMs and their target genes and interpreted their impacts on cancer pathogenesis. We found that there were significant differences in the target gene expressions between samples with and without the mutations. These results suggest that the binding of TFs to the candidate enhancer regions may be disrupted by these mutations, thus altering the expression of target genes. The protein product of the first example, TEAD1, is a member of the TEAD family of TFs. TEADs are involved in the Hippo signaling pathway, which regulates cell growth, proliferation and tissue homeostasis (34). Previous studies have demonstrated that *TEAD1* may be downregulated in renal, bladder, and certain types of breast cancers (34–36). The target gene of the second example is *CX3CR1*, which encodes a receptor for fractalkine (CX3CL1). CX3CR1 is mainly expressed in hematopoietic cells, natural killer cells, dendritic cells, T cells, monocytes and microglia (37,38). Higher expression of CX3CL1/CX3CR1 correlates with better prognosis and fewer recurrences in hepatocellular carcinoma (39). The target gene of the third example is *NFYB*, which encodes one of the components of NFY, a heterotrimeric transcriptional activator (40). NFY complex regulates cell-proliferation by controlling the expression of genes required for cell cycle progression. NFY also regulates cell survival through direct control of several anti-apoptotic genes (41). In summary, based on data analysis, we inferred the association between the CNRMs in the candidate enhancers and their possible target genes, which are suggested to be involved in cancer pathogenesis. Our findings suggest that these mutations in the three TFBSs could potentially serve as diagnostic markers.

Some of the remaining 20 994 (21 574 − 580) recurrent mutations, for which we could not assign their functional relevance in cancer pathogenesis and hence were defined as variants of unknown significance (VUS), may also be involved in cancer pathogenesis. To focus on *cis*-regulatory regions such as enhancer regions, we adopted stringent criteria to identify candidate CNRMs. Thus, some VUS may have an influence on cancer pathogenesis despite not fully meeting all the criteria used in this study. In addition, currently, the number of known non-coding cancer-related mutations is lower than those in coding regions. It has been reported that there are about 1200 cancer-related recurrent mutations in exonic regions, which comprise about 2% of the whole human genome (42,43). Assuming that the conserved non-coding regions, which comprise about 3% of the genome (44), have CNRMs at a frequency equivalent to those in exonic regions, more candidate CNRMs are likely to be found among the VUS in non-coding regions. Therefore, the functions of VUS need to be carefully validated using a combination of computational and experimental approaches. In addition, the development of new techniques for detecting chromatin interactions with high resolution and the accumulation of additional data on interactions between enhancers and promoters should provide new insight into cancer-associated mutations in non-coding regions.

Previous studies of cancer-related non-coding mutations have focused on functionally defined *cis*-regulatory elements such as promoters and enhancers (5,7,45–49). In these studies, various computer algorithms and pipelines for annotating recurrent mutations in non-coding regions were developed and several functional non-coding mutations were reported. For example, Hornshøj *et al.* identified non-coding mutations based on computational analysis of evolutionarily conserved sequences (8). In other studies, efforts were made to identify non-coding mutations in regions that might interact with gene promoter regions based on spatial arrangement of chromosomes using chromatin interaction data (46–49). In the Pan-Cancer Analysis of Whole Genomes project, a comprehensive identification of non-coding driver mutations was conducted using large-scale WGS data (22). They used a stringent filtering strategy based on sequence characteristics to remove false positives and artifacts, and identified highly confident candidate non-coding driver mutations. In contrast, we used mutation filtering strategies that are less stringent than those used in the previous study to identify cancer-related non-coding mutation candidates, followed by further filtering based on possible functions such as involvement in TFBSs and chromatin interactions. We combined data from several public databases, such as epigenomic and chromatin structure data, some of which had not been considered in these previous studies, and explored the cancer-related mutations throughout the entire non-coding region of the genome. Then, we evaluated the functions of the mutations based on the effects on gene expression.

In this study, we analyzed merged data from various cancer types due to the limited number of samples for each cancer type. Detailed understanding of cancer pathogenesis requires mutation analysis in distinct cancer types. Owing to the small sample size of currently available WGS data, such analyses could not be performed. In addition, we also used merged ATAC-seq data and 4DGenome data because a limited number and type of samples are available. It is possible that these data may not reflect exact conditions in individual cancer types. In the future, upon accumulation of WGS data and other associated data for various cancer types, it should be possible to identify non-coding mutations that are specifically associated with individual cancer types. Another limitation of our study is that, because we focused only on recurrent mutations in non-coding regions, we cannot rule out the possibility that other mutations as well as those found in this study affect the target gene expression levels.

Cancer development and progression is driven by mutations that are not only in coding regions, but also in non-coding ones. Accordingly, if the molecular mechanisms of cancer pathogenesis by non-coding mutations can be elucidated, such mutations would also become novel markers for diagnosis and targets for drug therapy. In conclusion, this study provides novel insights into the importance of non-coding recurrent mutations in cancer pathogenesis using a combination of currently available data.

## DATA AVAILABILITY

The results shown here are mainly based upon data generated by the TCGA Research Network (http://cancergenome.nih.gov/) and COSMIC (https://cancer.sanger.ac.uk/cosmic). Somatic mutation data in TCGA were derived from http://ideker.ucsd.edu/papers/wzhang2017/. The datasets used as filtering conditions during the current study are available in the Ensembl (https://asia.ensembl.org/Homo_sapiens/Info/Index), 4DGenome (https://4dgenome.research.chop.edu/), JASPAR (http://jaspar.genereg.net/), ENCODE (https://www.encodeproject.org/) and ChIP-Atlas (https://chip-atlas.org/).

## Supplementary Material

zcab008_Supplemental_Files
