# Supplementary material for: Pan-cancer analysis of non-coding recurrent mutations and their possible involvement in cancer pathogenesis
Source: NAR Cancer. 2021 Mar 22;3(1):zcab008. doi: 10.1093/narcan/zcab008 (PMC8210231; doi:10.1093/narcan/zcab008)
Supplement: zcab008_Supplemental_Files [file zcab008_supplemental_files.zip › figure_16_S.pdf]

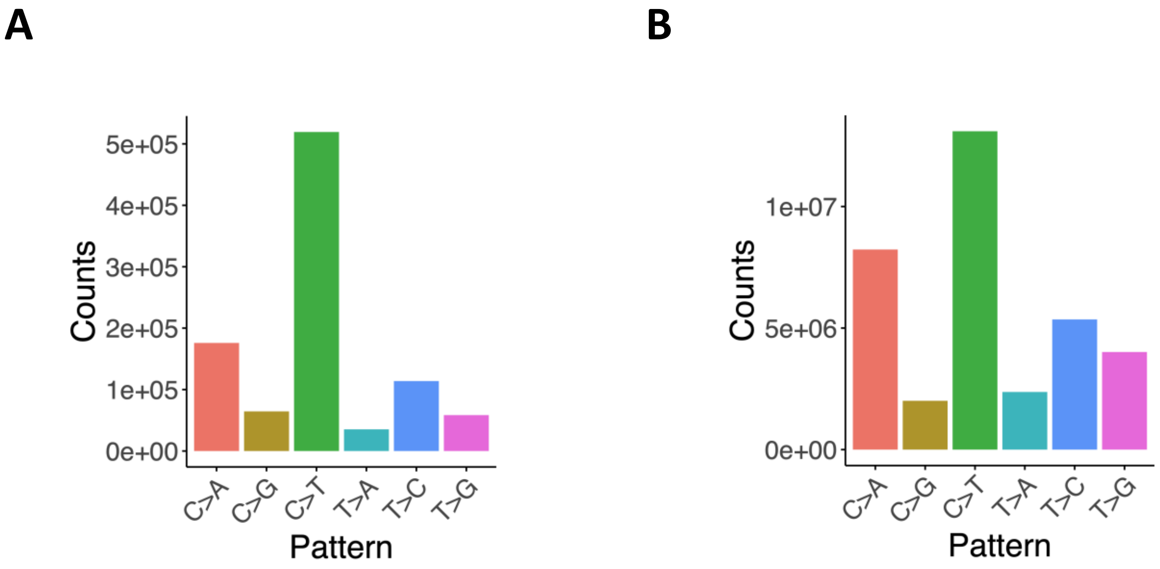

Supplementary Figure S1. The frequency of the six mutational patterns for those mutations from WGS data inTCGA.
(A) The mutations located in the coding regions. (B) The mutations located in the non-coding regions.

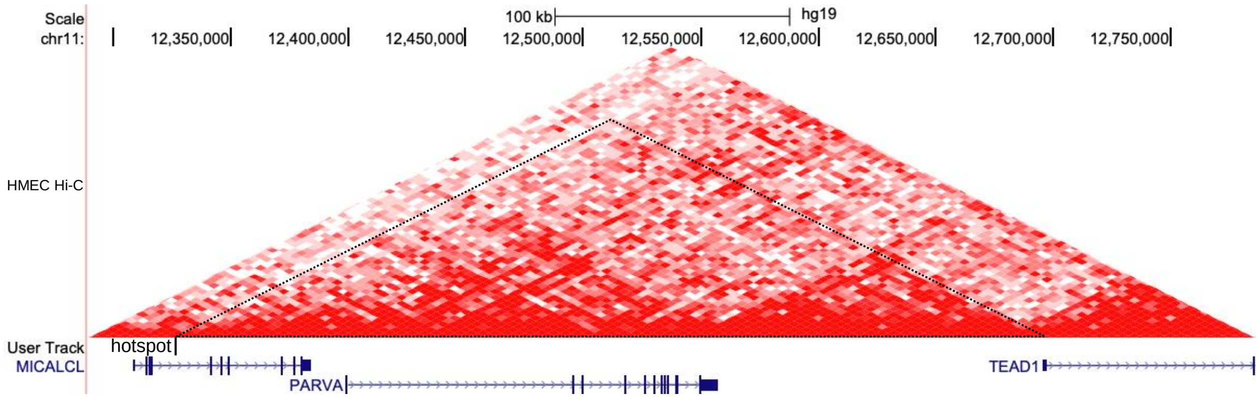

Supplementary Figure S2. Chromatin interaction between the RREB1 binding site with the recurrent mutation and *TEAD1*.

Upper triangle shows contact matrix determined by Hi-C in HMEC cells.

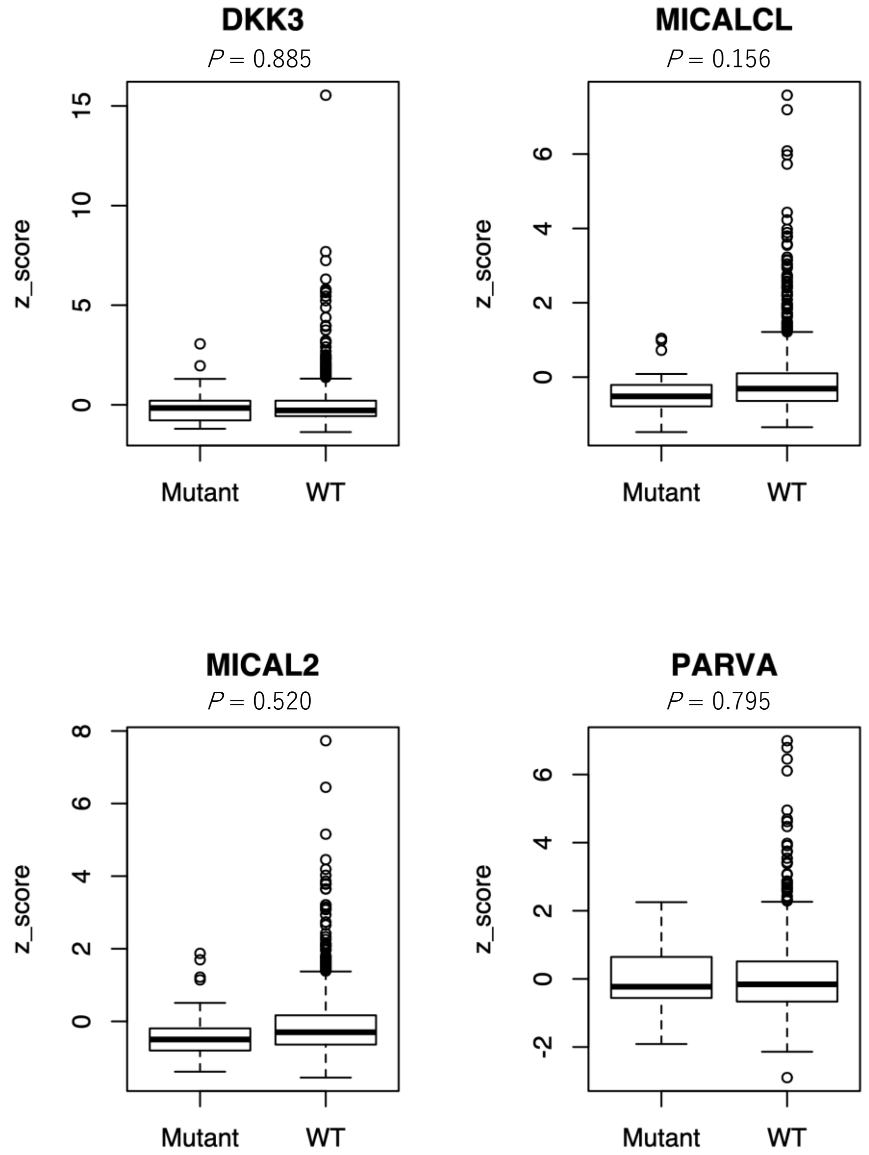

Supplementary Figure S3. Differential expression analysis of genes surrounding the RREB1 binding site.
"Mutant" and "WT" indicate samples with and without the recurrent mutation, respectively, in the RREB1 binding site.

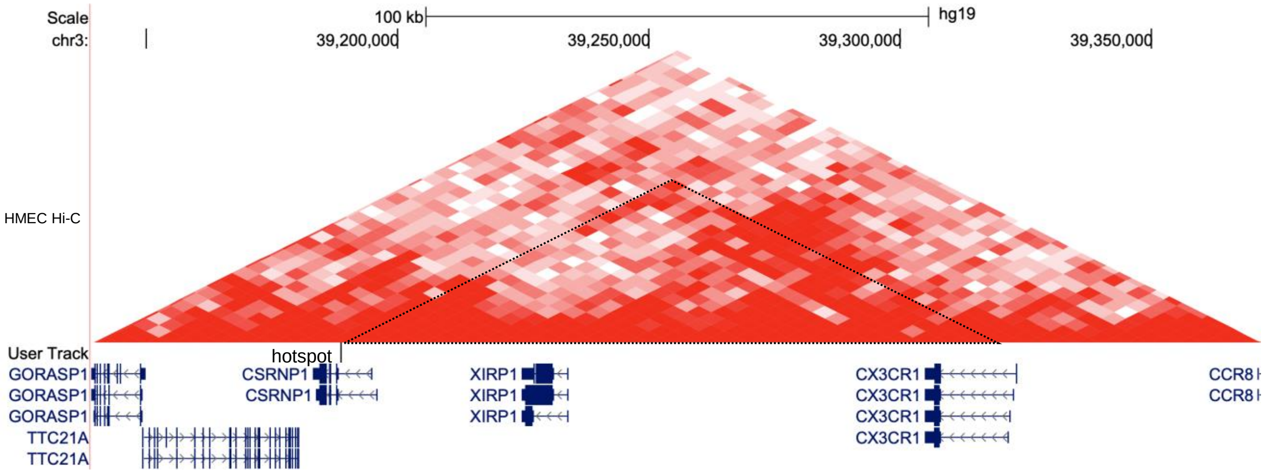

Supplementary Figure S4. Chromatin interaction between the ZSCAN4 binding site with the recurrent mutation and *CX3CR1*.
Upper triangle shows contact matrix determined by Hi-C in HMEC cells.

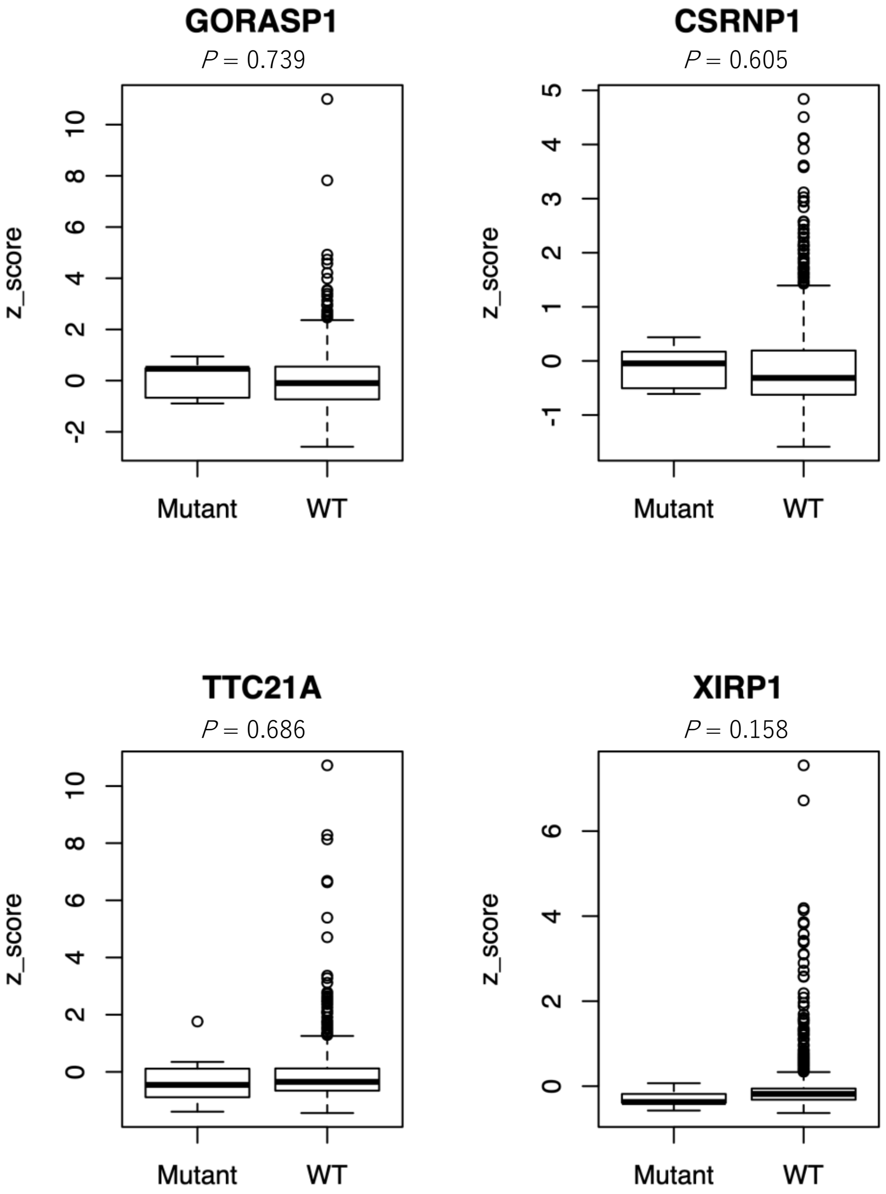

Supplementary Figure S5. Differential expression analysis of genes surrounding the ZSCAN4 binding site.
"Mutant" and "WT" indicate samples with and without the recurrent mutation, respectively, in the ZSCAN4 binding site.

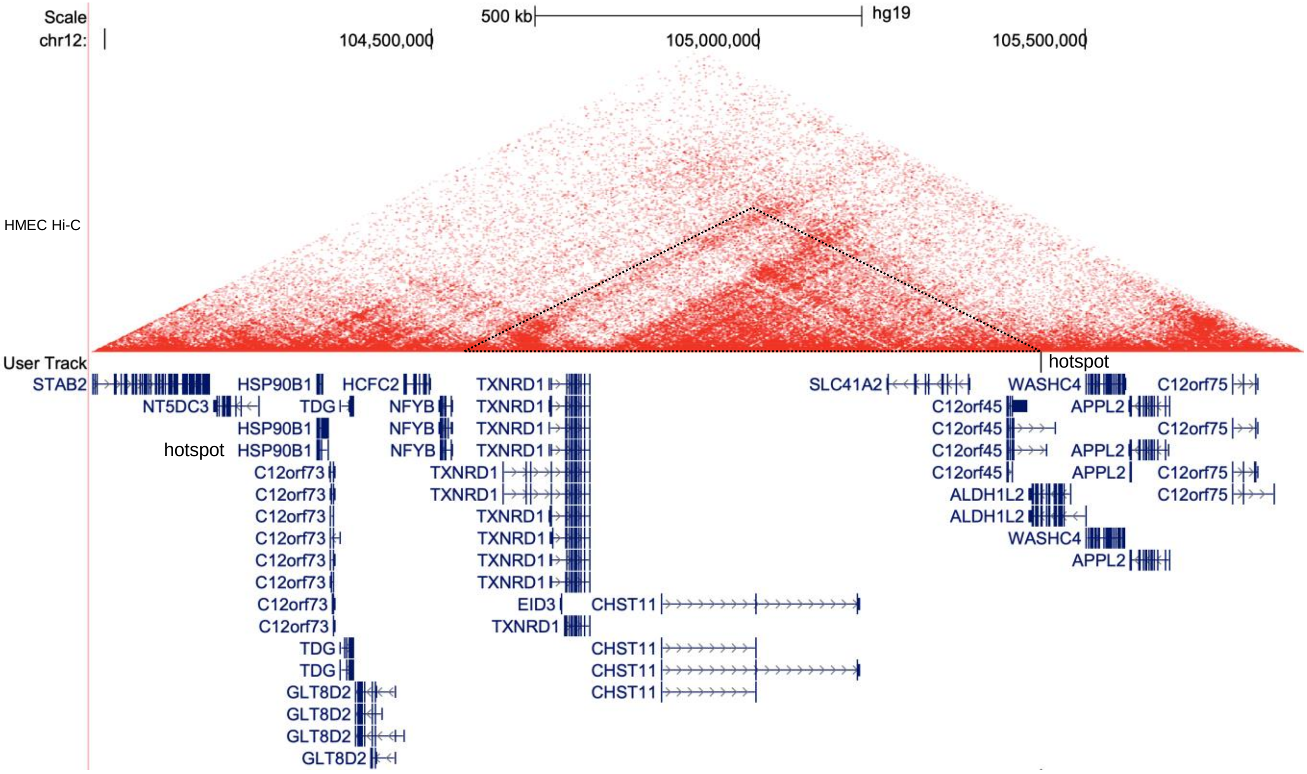

Supplementary Figure S6. Chromatin interaction between the RREB1 binding site with the recurrent mutation and *NFYB*.
Upper triangle shows contact matrix determined by Hi-C in HMEC cells.

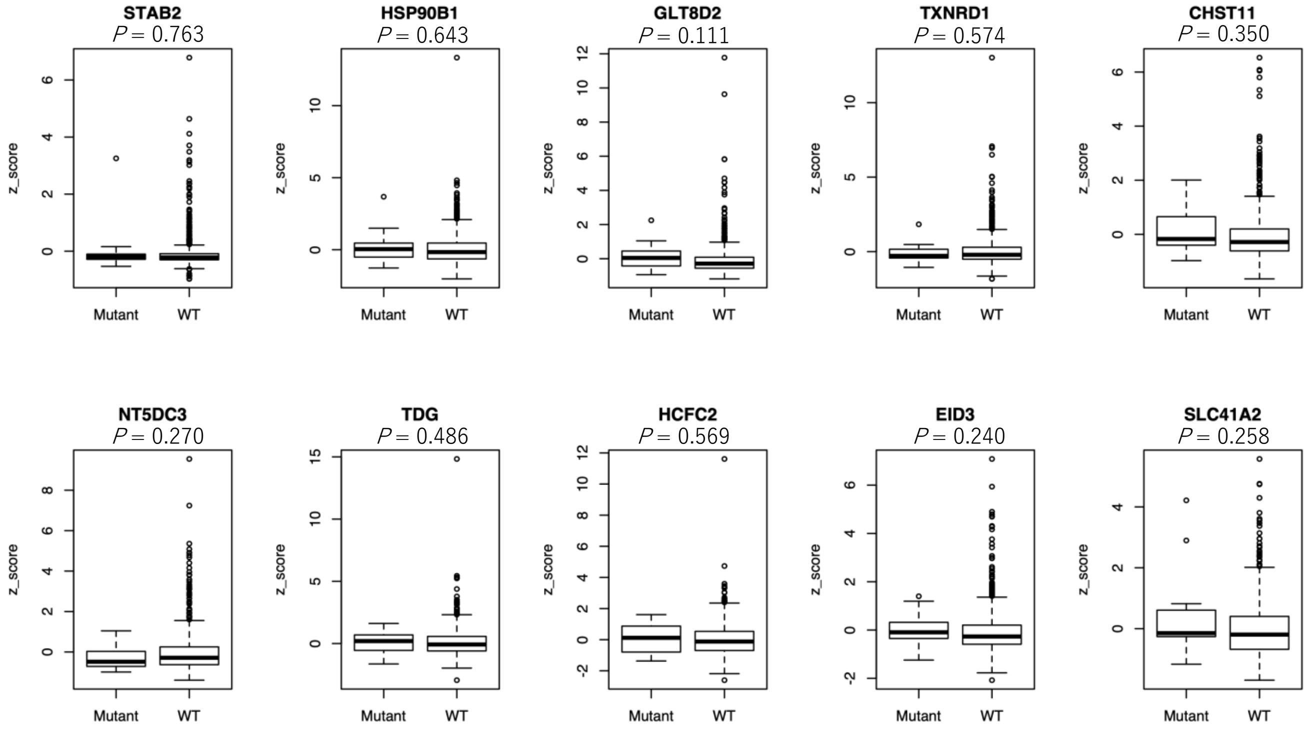

Supplementary Figure S7. Differential expression analysis of genes surrounding the RREB1 binding site.
"Mutant" and "WT" indicate samples with and without the recurrent mutation, respectively, in the RREB1 binding site.
